# Pea Protein for Hempseed Oil Nanoemulsion Stabilization

**DOI:** 10.3390/molecules24234288

**Published:** 2019-11-25

**Authors:** Maciej Jarzębski, Farahnaz Fathordoobady, Yigong Guo, Minghuan Xu, Anika Singh, David D. Kitts, Przemysław Łukasz Kowalczewski, Paweł Jeżowski, Anubhav Pratap Singh

**Affiliations:** 1 Faculty of Land and Food Systems (LFS), University of British Columbia, Vancouver Campus 213-2205 East Mall, Vancouver, BC V6T 1Z4 Canada; maciej.jarzebski@up.poznan.pl (M.J.); farah.fathordoobady@ubc.ca (F.F.); yigong.guo@ubc.ca (Y.G.); felixxu118@gmail.com (M.X.); anika.singh@ubc.ca (A.S.); david.kitts@ubc.ca (D.D.K.); 2 Department of Physics and Biophysics, Poznań University of Life Sciences, 38-42 Wojska Polskiego St., 60-637 Poznań, Poland; 3 Institute of Food Technology of Plant Origin, Poznań University of Life Sciences, 31 Wojska Polskiego St., 60-624 Poznań, Poland; przemyslaw.kowalczewski@up.poznan.pl; 4 Institute of Chemistry and Technical Electrochemistry, Poznan University of Technology, Berdychowo 4, 60-965 Poznań, Poland; pawel.jezowski@put.poznan.pl

**Keywords:** hempseed oil, pea protein, emulsion, stability, droplet size

## Abstract

In this paper, we present the possibility of using pea protein isolates as a stabilizer for hempseed oil (HSO)-based water/oil emulsions in conjunction with lecithin as a co-surfactant. A Box-Behnken design was employed to build polynomial models for optimization of the ultrasonication process to prepare the emulsions. The stability of the system was verified by droplet size measurements using dynamic light scattering (DLS) as well as centrifugation and thermal challenge tests. The z-ave droplet diameters of optimized emulsion were 209 and 207 nm after preparation and 1 week storage, respectively. The concentration of free Linoleic acid (C18:2; n-6) was used for calculation of entrapment efficiency in prepared nanoemulsions. At optimum conditions of the process, up to 98.63% ± 1.95 of entrapment was achieved. FTIR analysis and rheological tests were also performed to evaluate the quality of oil and emulsion, and to verify the close-to-water like behavior of the prepared samples compared to the viscous nature of the original oil. Obtained results confirmed the high impact of lecithin and pea protein concentrations on the emulsion droplet size and homogeneity confirmed by microscopic imaging. The presented results are the first steps towards using hempseed oil-based emulsions as a potential food additive carrier, such as flavor. Furthermore, the good stability of the prepared nanoemulsion gives opportunities for potential use in biomedical and cosmetic applications.

## 1. Introduction

Emulsion is a thermodynamically unstable state of the mixed phases which tend to rapidly separate. Two major categories of emulsion systems frequently used in food industry can be distinguished as: oil-in-water (O/W) and water-in-oil (W/O). There are many factors affecting the emulsion stability such as concentration, preparation techniques, presence of surfactants (stabilizers), temperature, storage conditions, etc. For the development of novel food and biomedical and pharmacological products, there is a need to use naturally-based stabilizers, such as plant extracts [1,2], proteins or hydrocolloids i.e., pectins [3], which present similar or better surface active properties than synthetic alternatives.

Proteins can play an essential role in forming and stabilizing the emulsion system thanks to their amphiphilic nature and film-forming abilities [4]. Different from the small molecular emulsifier which can rapidly diffuse to the interface during the emulsion formation, the proteins diffuse at a slower rate because of their high molecular weight [5]. In an emulsion matrix, proteins absorb to the oil/water interface and create compact layers around oil droplets, at the same time providing static electricity and spatial stability for the emulsion system [6,7]. The concentration of proteins dominates the oil droplet size [8]. Nowadays, the protein type emulsifiers are mostly derived from milk (or whey), soybean, egg, etc. because of their commercial availabilities, high nutritional values, and excellent functional properties [9]. However, these kinds of proteins are all defined as food allergens [10]. Thus, it is necessary to find consumer-friendly plant proteins to replace those from allergenic ones. Still, some of the valuable proteins sources, such as those obtained from the fraction of potato juice, have not been frequently used [11]. The limitations are related to the unsolved technological problems. Peas (*Pisum sativum* L.) are one of the most widely cultivated and exported legumes in Canada [12] and are recognized as a rich source of protein (18–30%) with a well-balanced profile of amino acids [13]. Due to allergenic properties of soyprotein, pea proteins with similar functional properties such as solubility, water- and oil-binding properties, viscosity, foaming, gelation, and emulsification, can be a promising alternative to soyproteins [14]. The emulsifying ability of different types of pea proteins as a solubility-dependent factor, was the least low at pH 5.0. However, the emulsifying property increased specially below this pH value, suggesting that these pea proteins have good potential as emulsifiers in acidic conditions [14,15]. Jiang et al. [16] showed that pea protein isolate exhibited good protection of the encapsulated vitamin D from UV light irradiation and improved antioxidant activity in pH-adjusted canola oil nanoemulsion.

Hempseed oil (HSO) extracted from the seed of *Cannabis sativa* L. is exceptionally valued for its nutritive properties associated with health benefits. The optimal ratio of ω-6 (linoleic acid, 18:2) to ω-3 (alpha-linolenic,18:3) fatty acids (2.1–3.1) in HSO ranges from 2.1 to 3.1 for balanced eicosanoid formation, which is linked to numerous physiological reactions in human body [17]. The presence of γ-linolenic acid (18:3); stearidonic acid (18:4); a noticeable amount of tocopherols and tocotrienols; phospholipids; carotenes; minerals as well as terpenoids and β-sitosterol also adds superior nutritional value to HSO compared to most seed oils [17,18,19]. The availability of the HSO is increasing due to the reintroduced demand for numerous products for cosmetic, nutraceutical, food, and functional food industries [20]. Only a few researches have actually focused on HSO emulsions. Thus, devoting more attention to the application of HSO in emulsion form, which might improve its beneficial properties and increase the stability of the oil, is needed.

One of the strategies to enhance the stability of emulsion systems is reduction of droplet size. McClements [21,22] suggested that nanoemulsion, which consists of small (d < 200 nm) oil droplets dispersed within an aqueous medium, can be useful for incorporating essential oils into a wide range of aqueous-based food products. The concentration of emulsifier and the method of preparation (high pressure homogenizers, sonicators, microfluidizers, etc.) greatly influence the oil droplet size [23]. Peng et al. [8] reported that oil droplet size of emulsion, flocculated state, and creaming stability of emulsions prepared by pea protein are also associated with temperature. Increased hydrophobic interactions in inter-droplet emulsions caused by applying heat treatment resulted in flocculation and more creaming stability [8]. Typically, high-pressure homogenizers, sonicators and microfluidizers are applied for nanoemulsion preparation [23]. For example, Mikulcova et al. [19] prepared nanoemulsion based on bioactive hempseed oil with non-ionic surfactants such as Span and Tween. Although investigations have generally mentioned that pea proteins have good potential to be used as an emulsifying agent, especially at acidic conditions, their application in food products are still limited.

This work aims to investigate the ability of pea protein as an emulsifier for preparing HSO emulsion. The Box-Behnken design method was applied for optimization of a two-step preparation process. Obtained nanoemulsions were tested according to their stability, droplet size, rheological properties, and color.

## 2. Results and Discussion

Using the optimum point values for various variables affecting the emulsion process can significantly improve the quality and properties of nanoemulsion. With an efficient optimization of these variables including concentration of protein and lecithin as well as ultra-sound process time, a HSO nanoemulsion with reasonable stability can be obtained. In this research, based on preliminary studies regarding factors affecting the properties of HSO including nanoemulsion particle size (PS), zeta potential (ZP) and polidispersity index (pdi), three independent variables of pea protein (0.2–1.4%), lecithin (0.0–6.0%) and time of ultra-sound process (0–20 min) were selected. Based on the Box-Behnken design, 15 runs of the experiment with two replications and three trials of the center points were tested. The results for PS, ZP and pdi responses are presented in Table 1.

### 2.1. Effect of Process Condition on Nano-Emulsion Properties

Applying an experimental design for process condition, response variables of hydrodynamic diameter z-ave (PS) and zeta potential (ZP) were recorded from 215–806 (nm) and −12.5–−27.3 (mV) with the pdi from 0.233 to 0.787 (Table 1). Using regression analysis and ANOVA and considering the significant probability (*p*-value, F-ratio) effects of nanoemulsion process variables on nanoemulsion properties, quadratic model equations with *R*^2^ from 92.93–94.34% were developed based on regression coefficients values to predict the responses of PS (nm), ZP(mV) and pdi (*y*_1_, *y*_2_ and *y*_3_) to independent variables of protein concentration (%), lecithin concentration (%), and time of process (min) (*x*_1_, *x*_2_ and *x*_3_).

*y*_1_ = 316.314 − 172.921*x*_3_ (1)

*y*_2_ = 2.571 − 3.200*x*_1_ + 2.552*x*_2_ − 3.583*x*_3_^2^ (2)

*y*_3_ = 0.443 − 0.189*x*_3_ − 0.0892*x*_2_^2^ (3)

**Figure 1:**
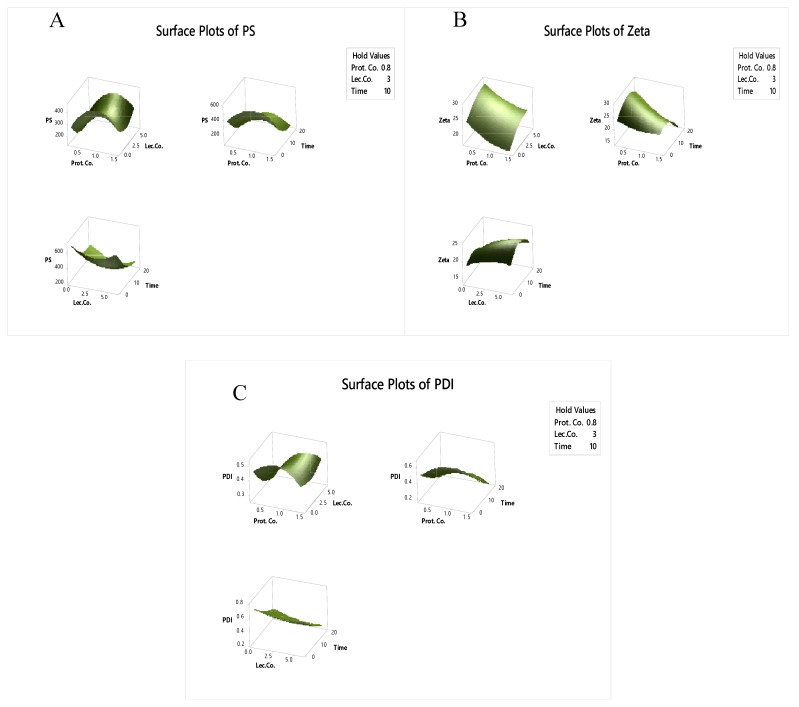
Response surface plot for *y*_1_ (**A**), *y*_2_ (**B**) and *y*_3_ (**C**): PS, ZP and pdi of the nanoemulsion in terms of protein (%), lecithin (%) and time of process (min).

It was found that the time of the ultrasound process had the most significant effect (*p* < 0.05) on all the response variables, especially on the particle size of emulsion. However, using different percentages of protein and lecithin showed no linear or quadratic effect (*p* > 0.05) on this response. Interaction effects of the process condition on all the emulsion properties were also found to be insignificant (*p* > 0.05). For better understanding the relation and effects of process factors on response variables, 3D graphical methodology are presented in Figure 1.

### 2.2. Optimization Procedure

Using graphical and numerical optimization for desired approaches, the optimum condition for the nanoemulsion process based on minimum hydrodynamic diameter z-ave (nm), polydispersity index (pdi) and maximum of zeta potential (mV), was developed. The overall optimal condition resulted in the highest amounts of zeta potential (−27.3 mV), and the smallest values for particle size and pdi (209 nm and 0.239, respectively) were predicted to be achieved at combined levels of 0.4% pea protein, 5.0% lecithin and 18.0 min ultra-sound with composite desirability of 0.985. The optimal levels of parameters were verified for the experimental responses as predicted by the mathematical model presented in Table 2. It can be seen that there was no significant difference between experimental and predicted values (*p* > 0.05) for all of the responses (error < 5.00%).

### 2.3. Optimized Emulsion Properties

Using the optimum conditions for preparing HSO emulsions resulted in 98.63% ± 1.95 and 92.72% ± 2.21 entrapment efficiency (based on the linoleic acid released in emulsion medium) for EMOP and EMO samples, respectively, indicating a significant effect (*p* < 0.05) of pea protein on HSO entrapment. One of the protein characteristics is surface hydrophobicity, resulting from hydrophobic groups present on the protein surface. These groups are partially denaturated proteins which provide greater potential for adsorption of oil at the oil/water interface [24].

#### 2.3.1. Particle Size and Zeta Potential Results

**Figure 2:**
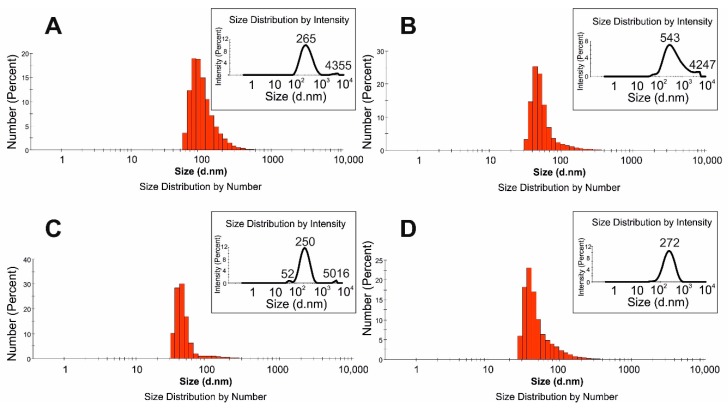
Particle size distribution obtained by DLS: (**A**), (**C**): EMOP; (**B**), (**D**): EMO after preparation and 1 week later respectively.

Particle and droplet size is one of the crucial parameters of emulsion stability. The stability of nanoemulsion system might be improved by the application of ultrasounds [25]. To increase the stability of HSO base emulsion, the ultrasounds process was used for preparation of the emulsion after the ingredients were mixed by a homogenizer. Then the emulsion droplet size was evaluated by DLS. Figure 2 shows the droplet size distribution of the optimized samples with (EMOP) and without (EMO) pea protein, obtained by DLS. Both the homogenization process and pea protein had significant impacts on final emulsion droplet size. Smaller droplet z-ave (209 nm) were observed in EMOP than EMO z-ave (309 nm). To make smaller droplets and increase the stability, the interfacial tension between the droplet must be decreased. Adding protein molecules such as pea protein to an emulsion system can result in decreasing interfacial tension, leading to lower Laplace pressure and smaller droplets. However, with a high concentration of protein, the interfacial elasticity of droplets decreases, resulting in less stability [26].

In both samples, no significant differences (*p* > 0.05) were observed in ZP results. Preliminary stability tests of the emulsions stored in a cold room at 4 °C were performed after 1 week. DLS results (Figure 2) showed that the maximum peak of droplet size distribution decreased from 265 to 250 nm and from 543 to 272 nm for EMOP and EMO, respectively. A decreasing droplet size during storage of the nanoemulsion has already been observed [27,28]. Badolato et al. [29] suggested that the less stable emulsion system possibly loses larger droplets (according to investigated storage conditions). Hence, detailed stability tests were also performed in the current study (see Section 2.4.).

#### 2.3.2. FTIR Results

**Figure 3:**
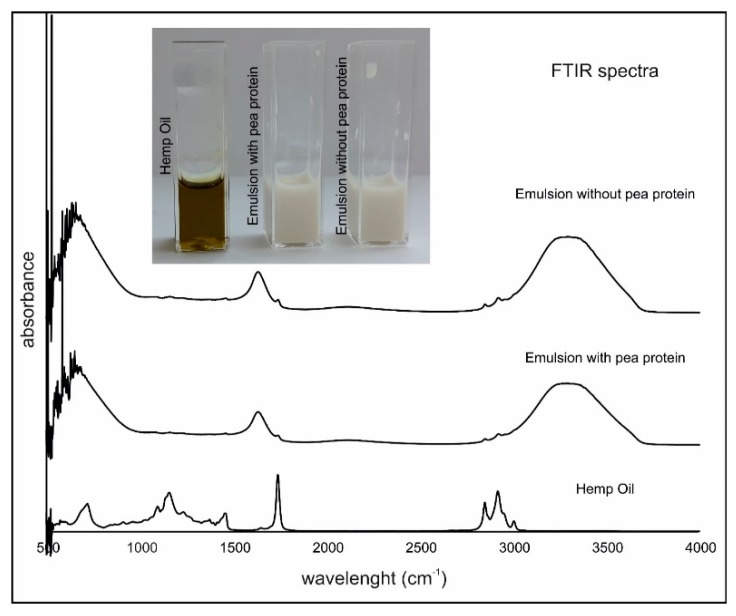
FTIR spectra and a sample image of hemp oil and hemp oil emulsions with and without pea protein.

Fourier transform infrared spectroscopy (FTIR) is one of the fastest spectroscopic methods for basic oil quality determination [30]. Figure 3 presents typical spectra obtained by FTIR for pure HSO from Canadian manufactured and two optimized emulsions (EMOP and EMO). There is no significant differences (*p* > 0.05) in spectra between samples with and without pea protein. Siano et al. [31] performed more detailed studies using Attenuated Total Reflectance-Fourier Transform Infrared (ATR-FTIR) of seeds and oils from edible fedora cultivar hemp (*Cannabis sativa* L.). They pointed out that signals registered around 1700 cm^−1^ belonged to a carbonyl band. Moreover, they indicated characteristic regions of *Cannabis sativa* L. at FTIR spectra for moisture, lipid, protein and carbohydrate. Our studies confirmed the presence of the strong signals at around 1700 cm^−1^. Furthermore, the signal was weaker in prepared emulsions. A broad signal occurred in the emulsions from 3200–3300 cm^−1^. It was noticed that some of the signals (peaks maxima) from HSO were relatively lower in the investigated emulsion. It might be caused by the low oil and high water concentrations, as well as the addition of surfactants in the emulsions.

#### 2.3.3. Rheological and Color Tests

**Figure 4:**
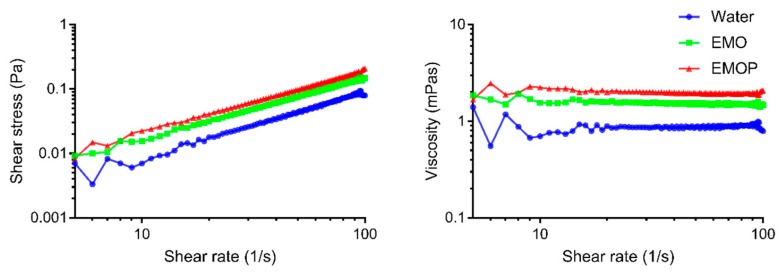
Flow curves of 10% hemp seed oil (HSO) O/W emulsions with or without pea protein as an emulsifier (EMO and EMOP).

Simple rheological tests on shear viscosity were performed to compare the behavior of prepared emulsion systems under continuous shear conditions. Results from rheological tests can provide valuable insight into the molecular interactions within the emulsion system, which may influence the stability as well as sensory properties. In conclusion, hempseed oil emulsions were showed to have water-like behaviors. Both EMOP and EMO emulsion systems had similar but slightly higher viscosity than water (Figure 4). The water-like behavior of hempseed oil emulsions was not significantly influenced by the addition of pea protein as a stabilizer. Besides, power law model (Ostwald de Waele model) was chosen to quantify the dependency of shear stress on shear rate by the power law indexes (*n*) because of its simplicity as well as the good fit to the data. Both EMO (*n* = 0.9598) and EMOP (*n* = 0.9583) showed near Newtonian behavior. These results indicated that the viscosity of samples were virtually independent from applied shear rates, which were in accordance with results reported by Demetriades et al. [32]. With low viscosity and near Newtonian behaviors, both EMO and EMOP can be more easily utilized in food production processes than other non-Newtonian fluids.

Moreover, it was shown that EMOP had slightly higher viscosities than EMO given the same volume fraction of the oil phase (Figure 4). The higher shear viscosity caused by additional pea protein can be accounted for by bridging flocculation of dispersing droplets. Other studies also confirmed that flocculated emulsions had higher viscosity. Flocculated droplets in emulsion systems are able to trap parts of the continuous phase, exhibiting a higher effective volume fraction of the dispersion phase. Therefore, with the same actual volume fraction phase, EMOP had slightly higher viscosities.

Simple CIE L*a*b* analysis for the evaluation of color properties of the prepared emulsions showed more lightness, slightly less green tonality and more yellow shifting for EMO (L/a/b:76.07/−2.28/10.31) than EMOP emulsion (L/a/b: 75.21/−2.44/9.94).

### 2.4. Stability of the Emulsion

For the optimized samples (EMOP and EMO), detailed stability tests were performed. The time and temperature dependence were investigated and verified by DLS. Alvarado et al. [33] suggested that DLS is favorable for small droplet size and mostly for oil-in-water emulsion systems and might be used for possible coalescence evaluation. In our studies, the prepared emulsion systems have been investigated using the procedure described in Section 2.3.1 in different periods of time. For the comparison of the results, the concentration of the samples was kept the same for all measurements.

**Figure 5:**
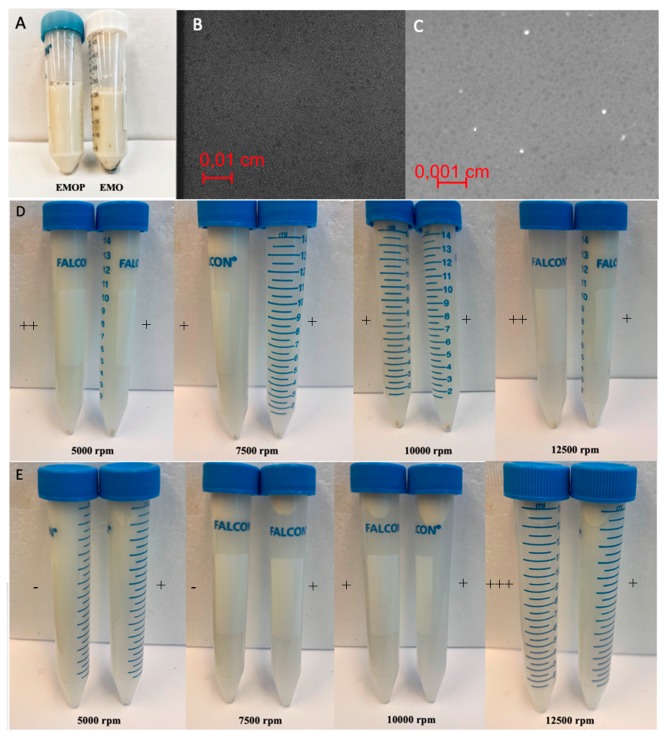
Images of emulsion with protein (EMOP) and without protein (EMO) during stability tests: (**A**) Images of EMOP and EMO after 2-week storage, (**B**) microscopic imaging of the sample emulsion in visible mode; 40×, (**C**) microscopic imaging of the sample emulsion in fluorescence mode; 40×, (**D**) sediment and creaming layer (**E**) of EMOP (left) and EMO (right) after centrifuging under certain rpm; scale: −: no visual effect; +: minimum effect, ++: medium effect, +++: maximum effect.

The results presented in Table 3 showed that after 1 week of storage, emulsion droplet size slightly decreased at room temperature and 50 °C. High emulsion droplet size stability at 50 °C suggested that a higher temperature has a preserving impact on nanoemulsion. An increasing droplet size and unpleasant smell were noticed in the samples stored at 37 °C (no detailed experiments were conducted). The homogeneity of the samples was evaluated using an optical microscope in visible and fluorescence mode. No coalescence of the droplets were observed. Moreover, a weak signal from the fluorescent part of oil was observed (Figure 5C), but no detailed studies were performed.

Furthermore, emulsion stability was monitored under gravitational and centrifugal forces. The emulsion with the protein (EMOP) showed better stability in both tests. Under the gravitational forces, sediments only occurred in the emulsion without the protein (EMO) after 2 weeks of storage (Figure 5). Generally, all emulsions are thermodynamically unstable. If they remain long enough, they will be separated into several layers, consisting of the top layer of oil droplets (cream) and the bottom water layer (serum). In order to test this phenomenon, centrifugal forces were used to accelerate this process. Under 5000 rpm, EMOP showed little sediments and no creaming layer while the EMO showed more sediment and a slight creaming layer (Figure 5). For the 7500 rpm test, EMOP still showed fewer sediments and no creaming layer. In contrast, the EMO had more sediments and an obvious creaming layer (Figure 5). Under 10,000 and 12,500 rpm, both EMOP and EMO showed a similar amount of sediments properly as they were both completely precipitated. However, EMO had a greater creaming layer compared to the EMOP (Figure 5). All the results showed that the EMOP had better stability compared to the EMO, which may result from more viscoelastic films formed by additional proteins.

Thermal resistance was tested based on three temperatures, which were room temperature, 37 °C and 50 °C. Particle size, zeta potential and entrapment efficiency were selected as three key factors to determine their thermal resistance. The temperatures were chosen according to their possible use in food, cosmetics or biomedical applications considering shelf-stable storage in room temperatures and possible temperature abuse in hot climates. The particle size and zeta potential of both EMO and EMOP were constant after 1 week of storage under room temperature and 50 °C, but significantly changed under 37 °C (Table 3). This was probably because 37 °C is more suitable for bacteria growth which might break down the emulsion system. The unpleasant smells of the samples after 1 week of storage under 37 °C confirmed this. All the EMOP samples also showed higher values for entrapment efficiency compared to EMO samples, suggesting less hempseed oil was released during 1 week of storage under three different temperatures (Table 3). The viscoelastic films formed by the protein can protect the oil phase in the particle and maximize the stability of the emulsion system.

The color analysis results for EMO and EMOP emulsion stored at room temperature, 37 °C and 50 °C showed no significant changes (*p* < 0.05) after 1 week of storage.

## 3. Materials and Methods

### 3.1. Materials

Unrefined cold-pressed hempseed oil (HSO) was purchased from Manitoba Harvest Hemp Foods (Winnipeg, MB, Canada). Refined lecithin was purchased from Alfa Aesar Co. Inc (Mississauga, ON, Canada). The pea protein was kindly given by Daiya Foods (Vancouver, BC, Canada). Other chemicals and solvents were of analytical or chromatography grade.

### 3.2. Sample Preparation

A two-step process was employed to prepare the O/W nanoemulsions. The pre-emulsions were prepared by a 1200W Polytron PCU-2-110 homogenizer (Brinkmann Instruments, Inc., Westbury, NY, USA). The preparing process was described as follows: 5 mL HSO was mixed with 45 mL distilled water. Then the different concentrations of lecithin and pea protein (shown in Table 1) were added into this solution and homogenized for 10 min at a speed setting of half of the full speed (rated at 10,000 rpm). Within 15 min, these pre-emulsions were ultrasonicated using a 200W UP 200ST (Hielscher Ultrasonics, Teltow, Germany) for a particular time determined by the experimental design using 40% of full power in continuous mode. To control the temperature during the ultrasonication process, all samples were kept in an ice-bath. All the nanoemulsions were stored at 4 °C for further tests.

### 3.3. Emulsion Characterization

#### 3.3.1. Droplet Size and Zeta Potential Determination

The hydrodynamic diameters (d_H_) of the emulsion droplets were obtained with dynamic light scattering (DLS) technique using Zetasizer Nano-ZSP (Malvern Panalytical Ltd., Malvern, UK). The measurements were performed at a detector angle of 173° and temperature of 25 °C. The average values from three measurements of particle size distribution were presented. Zeta potential was determined using Universal ‘Dip’ Cell Kit with Zetasizer Nano-ZSP (Malvern Panalytical Ltd., Malvern, UK) at 25 °C. All samples were diluted to the concentration of 1 mg/mL and all measurements were in triplicate, as well.

#### 3.3.2. Spectroscopic investigations FTIR

Infrared spectra were obtained using PerkinElmer Spectrum™ 100 FT-IR spectrometer (PerkinElmer Inc., Waltham, MA, USA). The experiments were performed using an HATR sampling accessory dedicated for liquid samples. Spectra were recorded in the rage from 370–7800 cm^−1^.

#### 3.3.3. Rheological Tests

Rheological tests were carried out using Modular Compact Rheometer MCR302 (Anton Paar GmbH, Graz, Austria). Measurements were performed at 25 °C using measuring plate PP50 (D:50 mm) and inset I-PP50/SS (D: 50mm; stainless steel cat. no. 16222). After the plate reached the adjusted temperature, 2 mL of samples were pipetted on the plate surface. The predefined templates from RheoCompass software (Anton Paar GmbH, Graz, Austria) for low-viscous samples were used for the test. The results from three replications were evaluated with RheoCompass software (Anton Paar GmbH, Graz, Austria). The GraphPad Prism v. 7.04 was used to treat the data.

#### 3.3.4. Colorimetry

For color evaluation, LabScan XE spectrophotometer (Hunter Associates Laboratory, Inc., Reston, VA, USA) equipped with EasyMatch QC software was applied. Before examinations, 5 mL of the sample was inserted into the transparent plastic small Petri dish-like plate. Whole measurements were repeated three times and average values ± SD were recorded.

#### 3.3.5. Fatty Acid Analysis (GC Method)

Fatty acid profile of the emulsions was determined according to the standard AOAC procedure [34] with some modification using a Shimadzu GC-17A Gas Chromatograph system (Shimadzu, Scientific Instruments, Inc., Columbia, MD, USA) equipped with a flame ionization detector (FID) and Shimadzu Class-VP Software. A fused silica capillary column (Omegawax™ 320, 30 m × 0.32 mm ID × 0.25 µm film thickness) was used for the separation. The initial temperature of the column was set at 165 °C for 10 min followed by increasing to 200 °C with a rate of 1.5 °C/min. Individual fatty acids were determined by comparison to the retention times of a mixture of fatty acid methyl ester (FAMEs) standard.

#### 3.3.6. Entrapment Efficiency (%)

Preliminary experiments were performed in order to find a reliable marker for determination of the entrapment or incorporation efficiency of HSO within the emulsion structure. It was found that linoleic acid (C18:2; *n*-6) as the most abundant fatty acid in HSO [17] could be considered as a marker for tracking the entrapment efficiency. For this purpose, the prepared samples were centrifuged at 12,000 rpm at 15 °C using Legend X1R centrifuge (Thermo Fisher Scientific, Inc., Waltham, MA, USA) for 15 min to sediment the nanoemulsion particles from the aqueous phase. When the emulsion suspension was centrifuged, the un-bond HSO was floated. Following centrifugation, the supernatant was collected and the fatty acid percentages including linoleic acid were determined according to the method described in 3.3.5. The entrapment efficiency (%) of linoleic acid (LA) concentration was determined by comparing the amount of LA incorporated into the emulsion structure with the initial amounts of LA in hempseed oil wherein the amounts of LA are based on the respective chromatogram peak area.

(4) EE (%)=((Total LA −Determined free LA)Total LA )×100

### 3.4. Emulsion Stability

#### 3.4.1. Stability Tests

The stabilities of emulsions with or without proteins (EMO and EMOP) were tested using two methods: simple and accelerated methods under gravitational and centrifugal forces, respectively. Both methods were followed by visual observation based on sedimentation, creaming or flocculation. The tested emulsions for the simple method were placed in 50-mL sealed transparent centrifuge tubes and kept for 14 days at room temperature (22 °C). The tested emulsions for the accelerated method were placed in 15-mL sealed transparent centrifuge tubes and diluted 10 times for visual proposes. Then, all the samples were centrifuged for 10 min under 5000, 7500, 10,000, and 12,500 rpm. All the accelerated tests were conducted with Sorvall Legend X1R Centrifuge Series (Thermo Fisher Scientific, Inc., Waltham, MA, USA).

#### 3.4.2. Thermal Resistance Studies

Heat resistance of emulsions with or without proteins (EMO and EMOP) were tested under three temperatures including room temperature, 37 °C and 50 °C. An amount of 5 mL of these emulsions were incubated at Sanyo MIR-553 (Sanyo, Japan) incubator for 1 week under the mentioned temperatures. Then, all the samples were tested for their particle size (nm), zeta potential (mV) and entrapment efficiency (%) as described before. All the tests were performed in triplicate.

#### 3.4.3. Microscopic and Macroscopic Investigations

The images of the emulsions were taken from Axio Observer.z1 (Zeiss, Oberkochen, Germany). The imaging was performed in fluorescence and light mode with different magnifications (10×/0.25 air, 40×/1.3 oil, 100×/1.45 oil). The images were processed using ZEN imaging software (Zeiss, Oberkochen, Germany). The stability and homogeneity of the samples were evaluated by eye observation during storage. To present the inhomogeneity, phase separation and easily distinguish color variation between samples, simple pictures were taken using Samsung J7’s built-in camera.

### 3.5. Experimental Design and Statistical Analysis

In the present study, a Box-Behnken design was employed to build polynomial models for process optimization using Minitab software version 18.0. The design consisted of three replications at center points to evaluate the pure error. To assess the main, interaction and quadratic effects of the process parameters including protein concentration (0.2–1.4%), surfactant concentration (0–6%) and sonication time (0–20) on response variables, a non-linear model was generated as follows:

*y* = *β*_0_ + *β*_1_*x*_1_ + *β*_2_*x*_2_ + *β*_3_*x*_3_ + *β*_4_*x*_1_*x*_2_ + *β*_5_*x*_2_*x*_3_ + *β*_6_*x*_1_*x*_3_ + *β*_7_*x*_12_ + *β*_8_*x*_22_ + *β*_9_*x*_32_ (5)

in which *y* is the response variables associated with the combination of each factor-level; *β*_0_–*β*_9_ are the coefficients of the regression for respective variables computed from the experimental values of *y*; and *x*_1_, *x*_2_, and *x*_3_ represent the independent variables. The experiments were randomized to decrease the unexplained effects of responses variability due to unimportant factors.

The numerical and graphical optimization were performed for finding the optimal condition of the nanoemulsion process for desirable values of response variables including particle size (nm), polydispersity index (pdi) and Zeta potential. For a better conception of the significant effects (*p* < 0.05), graphical optimization was performed by 3D plotting of the reduced response models. The mathematical model was verified by carrying out the experiment in the given optimal conditions and data analysis using T-test.

To define the significance of the difference between the experimental runs, data were subjected to one way of ANOVA. All of the test analyses were performed in triplicate and reported as the mean ± SD. The significant difference (*p* < 0.05) among means was determined by Tukey’s test

The experiment design matrix generated by the software is shown in Table 1.

## 4. Conclusions

Performed examinations on hempseed oil nanoemulsion proved the impact of pea protein on emulsion stability improvement. Applying a Box-Behnken design of the experiment for process conditions, followed by process optimization for preparing the nanoemulsion with pea protein resulted in obtaining more than 98.00% encapsulation efficiency with the smallest average particle size and pdi values (209 nm and 0.239) and desired zeta potential (−27.3 mV). The presented results showed that the prepared emulsion had a water-like rheological behavior. Both EMOP and EMO nanoemulsions had similar but slightly higher viscosity than water. This indicated that the water-like behavior of hempseed oil emulsions was not influenced by adding pea protein as a stabilizer. Based on the results of the stability tests, the EMOP showed better results for particle size, zeta potential and entrapment efficiency after 1 week of storage and centrifugal accelerating tests. This was caused by the fact that the protein can form viscoelastic films on the outer part of the droplets, protecting the oil phase in the particle and maximizing the stability of the emulsion system. In addition, obtained preliminary tests showed that the storage temperature of 37 °C might result in microbial growth. Further detailed investigations are needed for the possible use of hempseed oil nanoemulsion in food, cosmetic and biomedical applications.

## Figures and Tables

**Table 1:** Experimental design and responses results for nanoemulsion process of hemp seed oil.

Test Run	Nano-Emulsion Process Condition (Factors)			Response Variables		
	Pea Protein Conc. (%)	Lecithin Conc. (%)	Ultrasound Process Time (min)	Hydrodynamic Diameter z-ave (nm)	Zeta Potential (mV)	Poly-Dispersity Index (pdi)
1	1.4	3	20	220	−13.9	0.233
2	0.8	0	0	806	−18.2	0.787
3	0.8	6	0	803	−22.5	0.717
4	0.2	0	10	270	−24.3	0.440
5	1.4	6	10	314	−23.4	0.459
6	0.2	6	10	215	−27.3	0.261
7c	0.8	3	10	275	−24.3	0.405
8	1.4	3	0	414	−20.3	0.548
9	0.8	6	20	198	−21.6	0.324
10	1.4	0	10	372	−19.4	0.431
11	0.2	3	20	227	−26.9	0.249
12	0.8	0	20	286	−12.5	0.335
13c	0.8	3	10	332	−22.0	0.443
14	0.2	3	0	292	−24.1	0.475
15c	0.8	3	10	343	−24.4	0.483
EMO ^1^	0.0	5	18	306	−29.9	0.415
EMOP ^2^	0.4	5	18	209	−27.3	0.239

c: center point; ^1^ Optimized emulsion without protein; ^2^ Optimized emulsion with protein.

**Table 2:** Comparison of experimental and predicted values of PS, ZP and pdi using optimal levels of protein (0.4%), lecithin (5.0%), and process time (18 min).

Test	PS (nm)	ZP (mV)	pdi
Experimental	209 ^a^	−27.3 ^a^	0.239 ^a^
Predicted	204 ^a^	−27.9 ^a^	0.240 ^a^
Error (%)	2.39%	2.15%	0.41%

^a^ Values with same letters in the same column were not different significantly (*p* > 0.05).

**Table 3:** Stability tests: DLS and Encapsulation efficiency (EE%) results for emulsions without protein (EMO) and with protein (EMOP) after 1 week of storage at room temperature, 37 °C and 50 °C.

Sample	Storage Condition	DLS Analysis				EE (%)
		z-ave	pdi	ZP (mV)	MPM ^1^	
EMO	Room temperature	212 ± 1	0.219 ± 0.011	−27.2 ± 0.65	261 ± 24	90.4% ± 1.1
	37 °C	282 ± 2	0.370 ± 0.007	−21.8 ± 0.87	377 ± 36	87.5% ± 1.5
	50 °C	217 ± 2	0.227 ± 0.017	−35.5 ± 3.86	280 ±21	89.3% ± 1.3
EMOP	Room temperature	206 ± 2	0.188 ± 0.025	−42.1 ± 0.67	252 ± 20	98.6% ± 2.1
	37 °C	343 ± 1	0.205 ± 0.020	−18.2 ± 0.53	414 ± 15	96.3% ± 1.9
	50 °C	201 ± 1	0.203 ± 0.003	−29.1 ± 3.66	252 ± 7	96.9% ± 2.1

^1^ main peak maximum.
